# Artificial Intelligence-Driven Approaches to Managing Surgeon Fatigue and Improving Performance

**DOI:** 10.7759/cureus.75717

**Published:** 2024-12-14

**Authors:** Ayan Bin Rafaih, Kaso Ari

**Affiliations:** 1 Education, Aitchison College, Lahore, PAK; 2 Surgery, Norfolk and Norwich University Hospital, Norwich, GBR

**Keywords:** artificial intelligence, fatigue, performance, surgery, surgical skills

## Abstract

Surgeon fatigue significantly affects cognitive and motor functions, increasing the risk of errors and adverse patient outcomes. Traditional fatigue management methods, such as structured breaks and duty-hour limits, are insufficient for real-time fatigue detection in high-stakes surgeries. With advancements in artificial intelligence (AI), there is growing potential for AI-driven technologies to address this issue through continuous monitoring and adaptive interventions. This paper explores how AI, via machine learning algorithms, wearable devices, and real-time feedback systems, enables comprehensive fatigue detection by analysing physiological, behavioural, and environmental data. Techniques such as heart rate variability analysis, electroencephalogram monitoring, and computer vision-based behavioural analysis are examined, as well as predictive models that provide proactive solutions. These AI-driven systems could suggest personalized break schedules, task redistribution, and interface adaptations in response to real-time fatigue indicators, potentially enhancing surgical safety and precision. However, ethical challenges, including data privacy and surgeon autonomy, must be carefully navigated to foster acceptance and integration within clinical settings. This review highlights AI’s transformative potential in optimizing fatigue management and improving overall outcomes in the operating room​​​​​.

## Editorial

Surgeon fatigue has long been a silent factor influencing surgical outcomes. Historically, fatigue management strategies have relied on structured breaks, duty-hour limitations, and wellness initiatives. However, these strategies do not address real-time fluctuations in fatigue that can occur during long or complex operations. As a field, we are only beginning to acknowledge the profound impact fatigue has on surgical performance - slower reaction times, decreased cognitive sharpness, and diminished dexterity. In an era where technology continues to transform healthcare, artificial intelligence (AI) offers a new frontier for detecting and mitigating fatigue in real time. But the question remains: Can AI provide meaningful, real-time solutions to an age-old problem? And, more importantly, will surgeons embrace AI-driven interventions?

Understanding surgeon fatigue

Surgeon fatigue is not a new issue. It has been associated with increased rates of errors, prolonged operative times, and even higher mortality rates [1,2]. A scoping review identified fatigue to be a major contributing factor in medication administration errors and near misses [3]. The effects of fatigue on performance are well-documented: cognitive and motor function are impaired as levels of alertness decline, reaction times slow, and decision-making capacity diminishes [4].

While initiatives to reduce fatigue, such as limiting on-call hours, have had some success, they are not tailored to the individual. Fatigue is dynamic; it can fluctuate during a single procedure depending on its complexity, the surgeon’s condition, and external stressors. Yet, in many cases, fatigue is undetectable until mistakes have already been made. Traditionally, methods such as the Psychomotor Vigilance Task (PVT) or the Epworth Sleepiness Scale (ESS) have been used to assess fatigue levels in high-risk professionals [5-8]. However, these methods are intrusive and challenging to implement in the operating room. Moreover, these tools measure only limited aspects of fatigue, such as reaction time or sleepiness, and are not designed for continuous monitoring. The traditional solutions lack precision and fail to account for the real-time physiological changes that surgeons experience during high-stakes operations. Here, AI presents an unprecedented opportunity to fill this gap. AI enables comprehensive monitoring by leveraging diverse data streams and advanced analytics. Unlike traditional methods, AI-driven systems can process data from multiple physiological sources (e.g., heart rate variability [HRV], electroencephalograms) in real time, providing an objective and continuous assessment of fatigue. This real-time capability is crucial in surgical settings, as it enables immediate intervention [9].

Role of AI in surgeon fatigue detection

Machine Learning Models for Fatigue Detection

Machine learning (ML) is central to AI-based fatigue detection. By training algorithms on physiological, behavioural, and environmental data, ML models can predict and classify fatigue levels with high accuracy [9-11]. Several studies highlight the use of ML models, such as support vector machines and convolutional neural networks (CNNs), for fatigue detection [12,13]. Research has shown that CNNs trained on electroencephalogram (EEG) data can detect neural fatigue patterns associated with decreased cognitive alertness [14]. EEG data captures brainwave patterns and has proven effective in indicating shifts in cognitive function related to fatigue. Similarly, unsupervised learning methods, such as clustering and anomaly detection, can identify unusual patterns in real-time data that may signify the onset of fatigue without requiring prior labelled datasets [15]. These models can be enhanced with reinforcement learning, which adjusts algorithms based on feedback, improving their accuracy over time as they adapt to individual fatigue patterns. By analysing these patterns, AI systems can predict fatigue onset and recommend pre-emptive interventions such as micro-breaks or shifts in task allocation.

Use of Wearable Technology

Wearable sensors are at the forefront of AI-driven fatigue detection, offering a non-intrusive way to collect real-time physiological data. Sensors embedded in wristbands, smart clothing, or surgical attire can monitor various biomarkers, including HRV, skin conductance, and muscle activity. By utilizing AI algorithms, these devices can provide real-time data on cognitive load and physical fatigue, flagging dangerous fatigue levels before errors occur [16-19]). HRV, a well-studied indicator of mental and physical fatigue, has been shown to decrease with increased stress and fatigue [20,21]. By using AI to analyse HRV patterns, wearable devices can provide a continuous stream of data for fatigue assessment. Additionally, skin temperature and galvanic skin response, which indicate stress levels, can offer insights into the physical and emotional state of the surgeon, further informing the AI’s assessment of fatigue levels [22]. One application of wearable technology is a smart wristband used in a study by Pimentel et al. [18], which monitored HRV in the operating and assistant surgeon. The study found that AI-driven analysis of these metrics was able to predict high levels of stress in the operating surgeon and mental fatigue in the assistant surgeon during intracranial aneurysm surgery, demonstrating the potential of wearables in high-stakes clinical environments.

Video Analysis for Behavioural Indicators

Computer vision, a branch of AI focused on analysing visual data, has shown promise in detecting behavioural indicators of fatigue [23]. Facial recognition algorithms can detect subtle changes in facial expressions, such as drooping eyelids, reduced blink rate, or prolonged eye closure, all of which are correlated with fatigue [24,25]. Additionally, eye-tracking technology, commonly used in driver fatigue detection, has shown potential in surgical settings, allowing AI systems to monitor attentional shifts or signs of drowsiness [26]. Siripurapu and Sataloff utilized voice to detect fatigue via AI [27]. The AI detected signs of fatigue with a high degree of accuracy, suggesting that video monitoring could be integrated into fatigue management systems in the operation room. This approach not only allows for continuous monitoring but also reduces the need for wearable devices, which some surgeons may find intrusive.

Integrating Environmental Data

Environmental factors, such as room temperature, lighting, and noise levels, can exacerbate surgeon fatigue. AI models can analyse environmental data in conjunction with physiological and behavioural indicators to predict fatigue levels more accurately. AI systems can incorporate these factors to provide a holistic view of fatigue triggers. For instance, inadequate lighting has been shown to increase eye strain and mental fatigue, while excessive noise in the operating room can lead to cognitive overload, further contributing to fatigue [28,29].

AI as a Proactive Solution: Predicting and Mitigating Fatigue

Beyond detecting fatigue, AI can offer proactive solutions. Predictive models using ML can analyse a surgeon's work schedule, past performance, and current physiological data to predict when fatigue is most likely to occur. These insights allow for personalized break schedules or even task redistribution during surgeries, where robotic systems take over repetitive or less complex parts of the procedure. Imagine a scenario where an AI system identifies a drop in a surgeon’s cognitive function based on reaction times or physiological changes. The system could suggest a rest period or even temporarily adjust the visual complexity of the surgical console to reduce cognitive load, helping the surgeon focus on critical tasks. This concept of adaptive interfaces represents a new way AI could directly assist surgeons in real time.

Challenges and ethical considerations

Privacy and Data Security

The collection and processing of biometric data raise privacy concerns, particularly regarding unauthorized access to sensitive health information. Strict data protection protocols and adherence to regulations, such as the General Data Protection Regulation (GDPR), are essential to protect surgeons’ privacy. Failure to secure data could lead to ethical and legal consequences [30].

Bias and Model Generalizability

AI algorithms trained on data from a homogenous sample may not generalize well across diverse populations. Physiological responses to fatigue vary based on factors such as age, gender, and ethnicity. To mitigate this, AI developers should use representative datasets and conduct validation studies across diverse demographic groups to improve model accuracy.

Surgeon Autonomy and Acceptance

The integration of AI in surgeon fatigue management raises significant ethical concerns. Fatigue management is traditionally left to the surgeon's discretion, with most relying on their own judgment to gauge when they are too fatigued to proceed. Will surgeons trust AI to tell them when they need a break? Or could they feel undermined by the idea of a machine monitoring their performance and intervening? Autonomy is a key issue here. AI-driven fatigue detection must be designed to support rather than replace the surgeon's decision-making. AI should serve as a guide, providing valuable insights and recommendations without infringing on the surgeon’s ultimate authority in the operating room. Trust will be critical in ensuring that these systems are embraced rather than resisted. Some surgeons may perceive AI monitoring as intrusive, potentially undermining their autonomy. Effective implementation of AI in the operating room requires engagement with surgical teams and a transparent approach that emphasizes AI as a support tool rather than a replacement for human judgment.

Future of AI in surgical fatigue management

Looking forward, AI has the potential to revolutionize how we approach surgeon fatigue. We are moving toward a future where AI-driven systems could intervene proactively, ensuring not just that surgeons take necessary breaks but that entire procedures are dynamically adjusted to accommodate fatigue levels in real time. This could lead to shorter recovery times for patients, fewer surgical complications, and a more sustainable working environment for surgeons. Yet, there is much work to be done. Clinical validation of these AI systems is essential, and cross-disciplinary collaboration between surgeons, AI developers, and ethicists will be crucial to ensure that AI’s potential is fully realized without sacrificing surgeon autonomy. For AI to become a trusted ally in the operating room, we need to build systems that surgeons not only use but also trust. The future is bright, but it will require thoughtful integration of AI technologies that are built on transparency, accuracy, and respect for the human element in surgery.

Surgeon fatigue is a critical issue that affects both surgical performance and patient outcomes. While traditional methods to manage fatigue are useful, they are insufficient for real-time monitoring and mitigation. AI offers a promising solution, with technologies such as wearable devices, cognitive load measurement systems, and real-time feedback platforms already showing success in fatigue detection. However, the successful integration of AI into surgical workflows will require addressing issues of autonomy, trust, and data privacy. The ongoing collaboration between AI developers, surgeons, and human factors researchers will be essential to refining these technologies and ensuring their ethical deployment in surgical environments.
